# The Metabolomic Bioenergetic Signature of *Opa1*-Disrupted Mouse Embryonic Fibroblasts Highlights Aspartate Deficiency

**DOI:** 10.1038/s41598-018-29972-9

**Published:** 2018-08-01

**Authors:** Cinzia Bocca, Mariame Selma Kane, Charlotte Veyrat-Durebex, Stéphanie Chupin, Jennifer Alban, Judith Kouassi Nzoughet, Morgane Le Mao, Juan Manuel Chao de la Barca, Patrizia Amati-Bonneau, Dominique Bonneau, Vincent Procaccio, Guy Lenaers, Gilles Simard, Arnaud Chevrollier, Pascal Reynier

**Affiliations:** 1 Equipe Mitolab, Institut MITOVASC, CNRS 6015, INSERM U1083, Université d’Angers, Angers, France; 2 Département de Biochimie et Génétique, Centre Hospitalier Universitaire, Angers, France; 3 INSERM U1063, Université d’Angers, Angers, France

## Abstract

OPA1 (Optic Atrophy 1) is a multi-isoform dynamin GTPase involved in the regulation of mitochondrial fusion and organization of the cristae structure of the mitochondrial inner membrane. Pathogenic *OPA1* variants lead to a large spectrum of disorders associated with visual impairment due to optic nerve neuropathy. The aim of this study was to investigate the metabolomic consequences of complete OPA1 disruption in *Opa1*^−/−^ mouse embryonic fibroblasts (MEFs) compared to their *Opa1*^+/+^ counterparts. Our non-targeted metabolomics approach revealed significant modifications of the concentration of several mitochondrial substrates, i.e. a decrease of aspartate, glutamate and α-ketoglutaric acid, and an increase of asparagine, glutamine and adenosine-5′-monophosphate, all related to aspartate metabolism. The signature further highlighted the altered metabolism of nucleotides and NAD together with deficient mitochondrial bioenergetics, reflected by the decrease of creatine/creatine phosphate and pantothenic acid, and the increase in pyruvate and glutathione. Interestingly, we recently reported significant variations of five of these molecules, including aspartate and glutamate, in the plasma of individuals carrying pathogenic *OPA1* variants. Our findings show that the disruption of OPA1 leads to a remodelling of bioenergetic pathways with the central role being played by aspartate and related metabolites.

## Introduction

OPA1 is a multi-isoform dynamin GTPase, mainly regulated post-translationally, that plays a central role in processing the mitochondrial inner membrane and in mitochondrial dynamics. Through its role in mitochondrial fusion, OPA1 intervenes in pleiotropic functions such as maintaining the integrity of mitochondrial cristae, apoptosis^1,2^, oxidative phosphorylation and energy production^3^, calcium fluxes^4^, mitochondrial DNA maintenance^5–7^, mitochondrial autophagic flux and renewal^8–10^, oxidative stress^11^, inflammation^12^, ageing^9,12^, neurogenesis^13,14^, and more generally, in mitochondrial plasticity and quality control^15^.

More than three hundred pathogenic variants have been reported in the *OPA1* LOVD (Leiden Open Variation Database) database^16^ since the first reports in 2000^17,18^. Pathogenic *OPA1* variants are responsible for a large spectrum of neurological disorders leading to visual loss, ranging from isolated optic neuropathy (DOA, Dominant Optic Atrophy, Kjer type, MIM(Mendelian Inheritance in Man)#165500) to severe multisystemic syndromes such as DOA associated with neurosensorial deafness (DOAD), the DOA+ syndrome (MIM#125250), and the early-onset Behr syndrome (MIM#210000)^19^. Most of these OPA1-related diseases follow an autosomal pattern of inheritance through a mechanism of haplo-insufficiency or a dominant negative effect. However, some rare diseases follow a biallelic pattern of inheritance involving pathogenic variants in association with hypomorphic variants^20^.

To date, very few OPA1 dysfunctions have been investigated by omics approaches. Our targeted metabolomic study on 9 tissues of the *Opa1*^*delTTAG/*+^ mouse model^9^ revealed a discriminating pre-symptomatic metabolomic signature in the optic nerves^21^. Apart from the blood, no other tissue showed this type of metabolic signature, indicating that OPA1 dysfunctions target mainly the retinal ganglion cells of the optic nerve. The pre-symptomatic optic nerve signature was characterized by decreased concentrations of sphingomyelins and lysophosphatidylcholines, suggestive of myelin sheath alteration, and by the modified concentrations of metabolites involved in neuroprotection or neurotoxicity, i.e. a reduction on dimethyl-arginine, carnitine, spermine and spermidine, and an increase of carnosine and glutamate, suggesting concomitant axonal dysfunction and excitotoxicity.

The same targeted metabolomic approach used for investigating 188 metabolites did not highlight any dominant metabolomic signature in fibroblasts from *OPA1* patients (n = 14) compared to controls (n = 8), in contrast to a study on another form of mitochondrial inherited optic neuropathy, i.e. Leber’s Hereditary Optic Neuropathy, which revealed a specific signature under the same experimental conditions^22^. This discrepancy may be explained by the relatively mild metabolic impact of the heterozygous pathogenic *OPA1* variants despite significant impairment of energy production and disruption of the mitochondrial network in fibroblasts from DOA patients^3^.

We used the recently developed non-targeted metabolomics pipeline for the identification of metabolites^23^ to investigate the plasma of patients with various OPA1-related phenotypes and pathogenic variants (n = 25) in comparison to healthy controls (n = 20)^24^. A robust predictive model characterizing OPA1 individuals was obtained, revealing alterations of the purine metabolism with an increase of inosine and a decrease of xanthine and hypoxanthine, related to the GTP/ATP (Guanosine Triphosphate/Adenosine Triphosphate) metabolism, as well as other metabolic alterations with a reduce concentration of urocanate, choline, glycerate, 1-oleoyl-rac-glycerol, rac-glycerol-1-myristate, aspartate and glutamate, and a rise in phosphocholine and in cysteine.

For a better analysis of the impact of complete OPA1 disruption on the whole cell and to further characterize its metabolic imprint, in this study we have used a null *Opa1* cellular model with a non-targeted metabolomic approach on *Opa1*^−/−^ mouse embryonic fibroblasts (MEFs), comparing the results with those of *Opa1*^+/+^ controls.

## Results

### Characterization of *Opa1*^−/−^ MEFs

**Figure 1:**
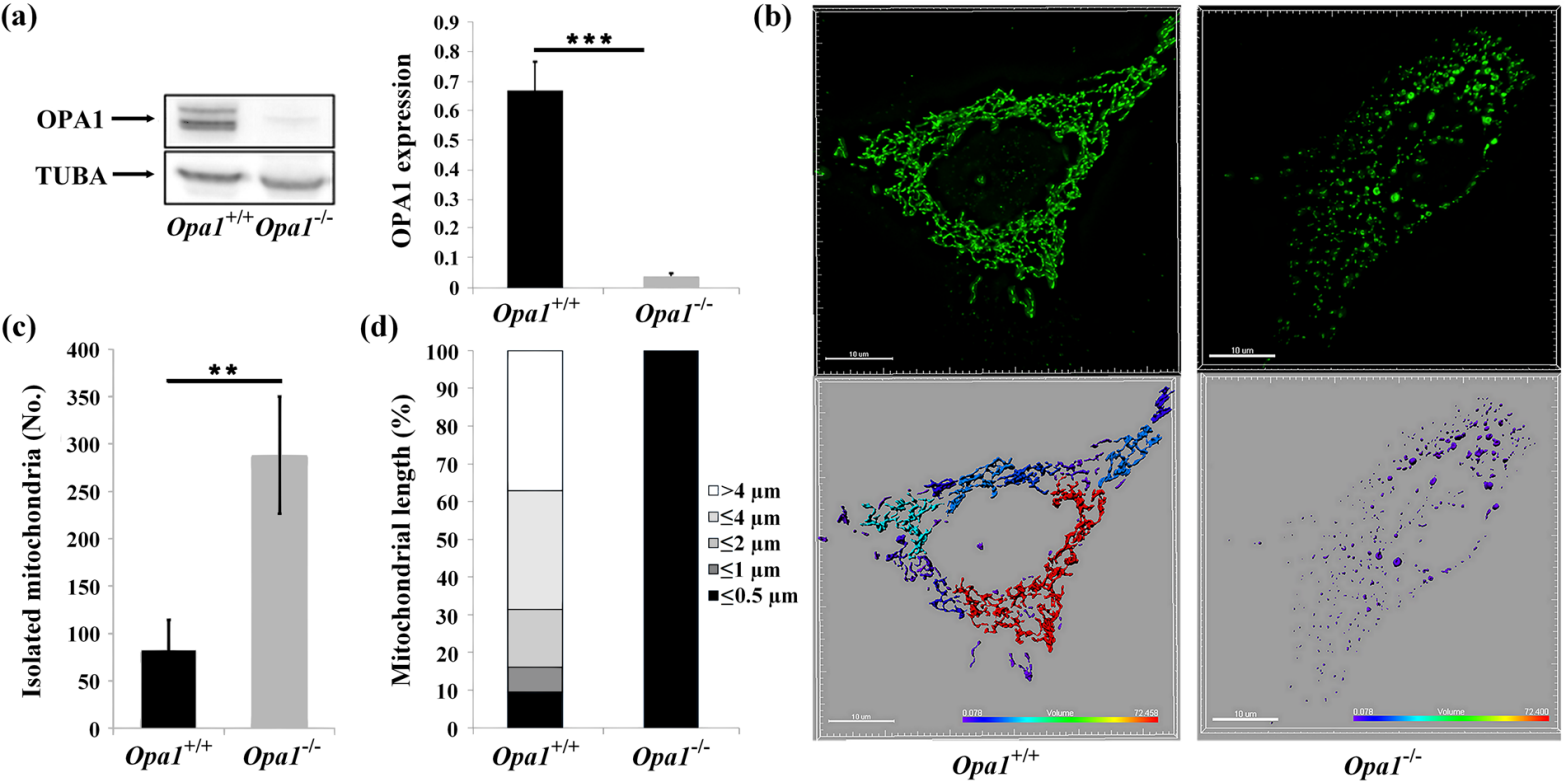
Molecular and cellular characterisation of *Opa1*^−/−^ and *Opa1*^+/+^ MEFs. (**a**) Representative western blot images. Thirty μg of protein from cell lysates were loaded on the gel and revealed after transfer by OPA1 and TUBA (Tubulin alpha) antibodies. Quantitative data were inferred by densitometric analyses of the immune-reactive bands. Histograms represent means ± SD of five independent experiments. The statistical analysis was carried out using non-parametric Mann-Whitney test, ***p-value < 0.001. The displayed blots were cropped, for full-length gels, see Supplementary Fig. S1. (**b**) Morphology of the mitochondrial network in *Opa1*^−/−^ and *Opa1*^+/+^ MEFs. Mitochondria were stained using Mitotracker Probes. Representative images of the mitochondrial distribution in *Opa1*^−/−^ and *Opa1*^+/+^ MEFs are shown. The mitochondrial network was modeled in 3D using Imaris software (Bitplane) and mitochondrial lengths were assessed and colour-coded. Scale bar = 10 µm. (**c**) Histograms show the number of isolated mitochondria per cell in each cell type. Statistical analysis was determined by Student’s unpaired *t-*test, **p-value < 0.01. (**d**) The bar graphs show the distribution of the mitochondrial population in five different categories on the basis of mitochondrial length.

**Figure 2:**
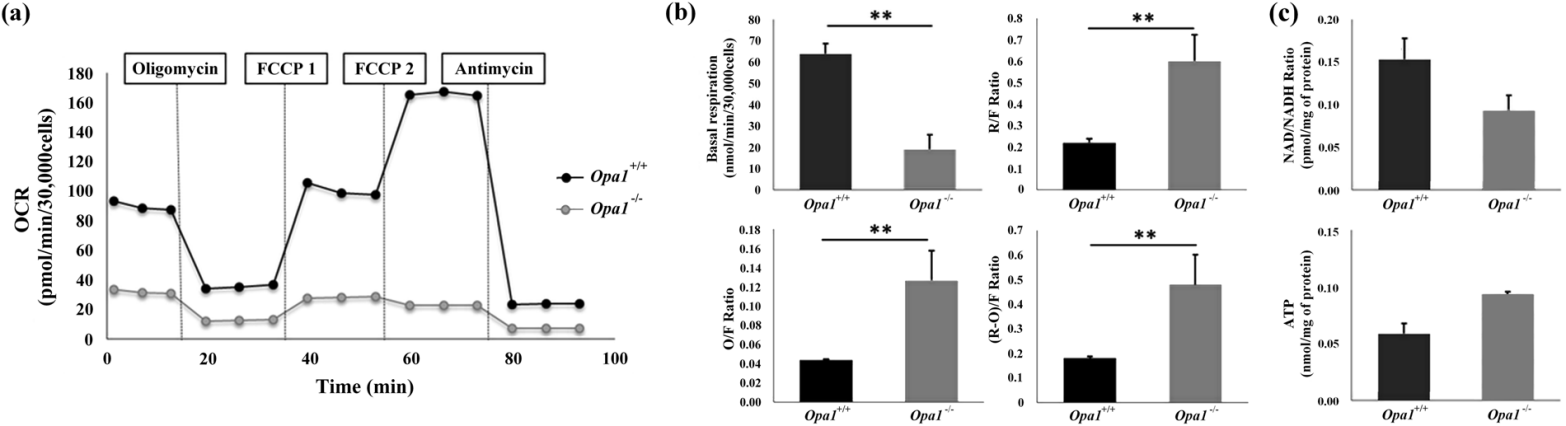
Mitochondrial respiration in *Opa1*^−/−^ and *Opa1*^+/+^ MEF. (**a**) Oxygen consumption rates were measured in both *Opa1*^−/−^ and *Opa1*^+/+^ MEFs with the Seahorse XFe96 extracellular flux analyzer. The OCR was evaluated with the following injection protocol: oligomycin (2 μg/mL), FCCP1 (0.25 μM) and FCCP2 (1.5 μM in this case) and antimycin A (2 μg/mL). (**b**) Basal respiration (R) represents the mitochondrial respiration sustained by endogenous substrates. Non-phosphorylating respiration (O) represents the residual respiration in the presence of oligomycin, whereas the maximal uncoupled stimulated respiration (F) was determined by titration of FCCP (0.25–3 μM). Values are expressed as respiratory control ratios, which in each case are inferred from the maximal uncoupled stimulated respiration (F). Histograms show the mean ± S.D values of four independent experiments. (**c**) NAD/NADH ratio and ATP in *Opa1*^−/−^ and *Opa1*^+/+^ MEFs incubated in glucose medium. Histograms show the mean ± S.D. of three independent experiments for the intracellular NAD/NADH and cellular ATP content. NAD, NADH and ATP values were normalised by protein concentration. The statistical analysis was carried out using Student’s unpaired *t‐*test (**p-value < 0.01).

Western blot analysis with whole-cell lysates of *Opa1*^−/−^ MEFs, compared to *Opa1*^+/+^ MEFs, confirmed the absence of OPA1 protein expression (Fig. 1a) (p = 0.0001). As expected, the lack of OPA1 led to complete fragmentation of the mitochondrial network, resulting in a higher number of isolated spherical mitochondria (+350%, *p* = 0.001, Fig. 1b,c). Seahorse XF96 respirometric analysis (Fig. 2) showed that in the resting state, the oxygen consumption rate of *Opa1*^−/−^ MEFs was significantly lower than that of *Opa1*^+/+^ MEFs (respectively, 19 (±*7.002*) *vs*. 64 (±*4.8*), *p* = 0.0015). The routine control ratio (R/F), which represents the fraction of the respiratory capacity used by cells to sustain respiration, and the phosphorylating control ratio (R-O/F), which reflects the fraction of the respiration used for ATP production under unstressed conditions, were both significantly greater in *Opa1*^−/−^ MEFs than in *Opa1*^+/+^ MEFs (respectively, 0.22 (±*0.0019) vs*. 0.60 (±*0.12)*, *p* = 0.0048 for R/F and 0.48 (±*0.08) vs*. 0.18 (±*0.07)*, *p* = 0.0029 for R-O/F). The leak control ratio (O/F) was also significantly greater in *Opa1*^−/−^ MEFs than in *Opa1*^+/+^ MEFs, accounting for 13% of the maximal uncoupled stimulated respiration compared to 4% in *Opa1*^+/+^ MEFs (*p* = 0.0364). Thus, consistently with earlier studies^25,26^, our findings show that the complete loss of OPA1 leads to mitochondrial fragmentation and mitochondrial respiration failure.

Other mitochondrial functional parameters such as in NAD/NADH and ATP were also assessed to evaluate the bioenergetics dysfunction. Our data shows an absence of alteration of NAD/NADH ratio (0.15 (±*0.02)* in *Opa1*^+/+^ *vs* 0.09 (±*0.019)* in *Opa1*^−/−^ (*p* = 0.0903)). Surprisingly, ATP content was not reduced in *Opa1*^−/−^ cells compared to *Opa1*^+/+^ (respectively, 0.09 (±*0.003*) *vs*. 0.05(±*0.01*), *p* = 0.1000); which could be accounted to higher glycolysis rates in these cells (Fig. 2c).

### Ninety MEF metabolites were accurately measured

**Figure 3:**
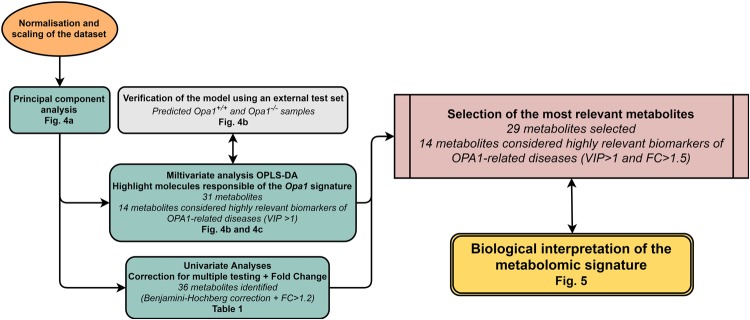
Statistical data analysis workflow chart. Each step of the statistical workflow chart is marked with the corresponding figure or table number in bold. The key elements summarizing the principal findings of this study are shown in italics.

The application of our data-analysis workflow chart (Fig. 3) led to the accurate detection of 90 metabolites in positive and negative ion modes. MS/MS fragmentation matching was possible for more than 80% of these metabolites. For the remaining 20%, the recognition of molecules was based on isotopic pattern compatibility with chemical formulae and retention times compared to those recorded in our in-house library. The most important classes of molecules detected were amino acids and secondary amines, other carboxylic acids, lipids, purines, pyrimidines and nucleotides, sugars and keto acids. The complete list of these metabolites, whether involved in the signature or not, is provided in Supplementary Table S1.

### The metabolomic signature of *Opa1*^−/−^ MEFs

**Figure 4:**
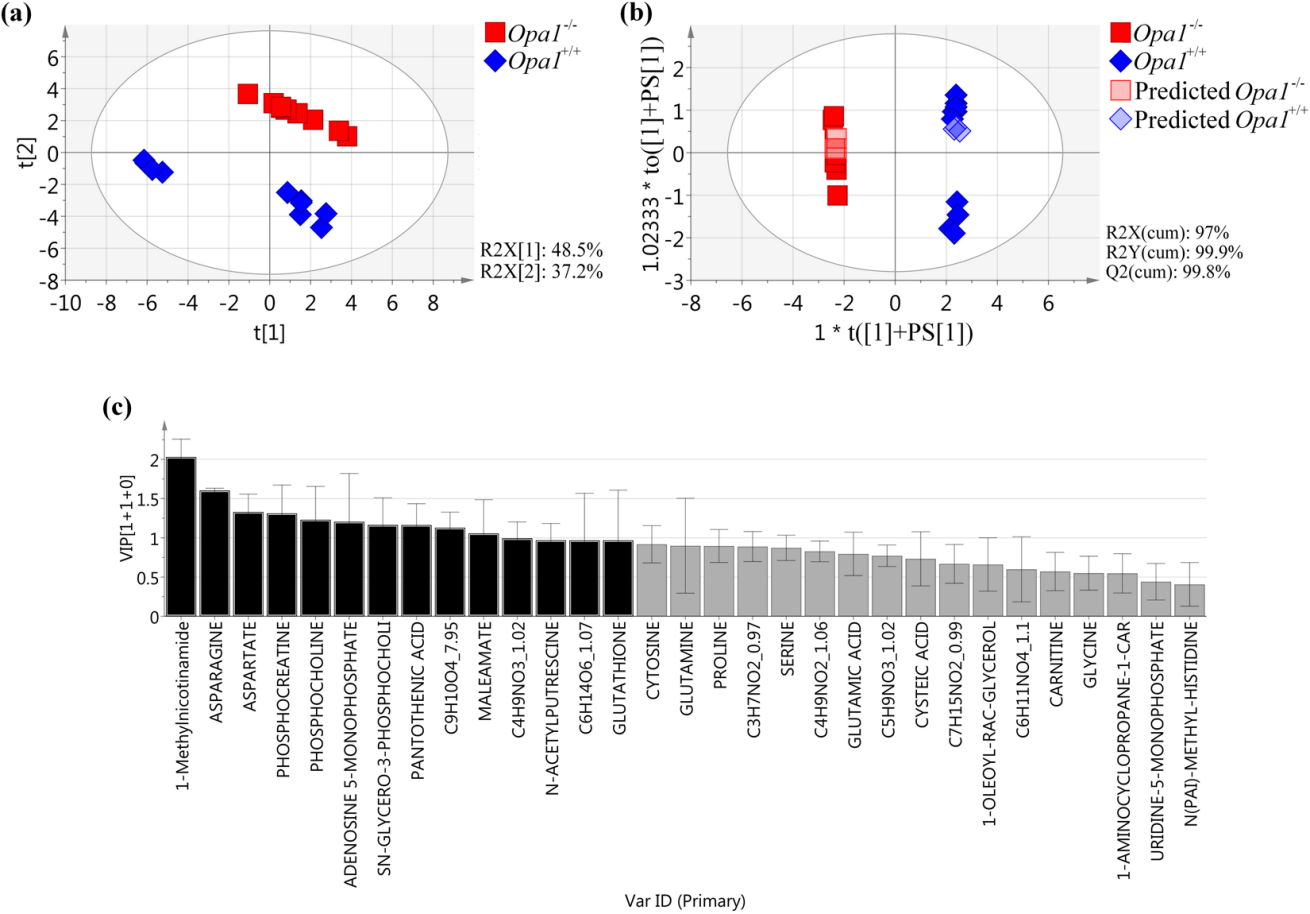
Metabolomic signature of *Opa1*^−/−^ *vs Opa1*^+/+^ MEFs. (**a**) Unsupervised PCA score plot of *Opa1*^+/+^ (blue rhombuses) and *Opa1*^−/−^ (red squares) MEF cell lines. There is a spontaneous separation on the t[2] axis related to genotype. (**b**) Supervised OPLS-DA score plot of *Opa1*^+/+^ (blue rhombuses) and *Opa1*^−/−^ (red squares) MEF cell lines with the prediction of test samples (predicted *Opa1*^+/+^ (light blue rhombuses) and predicted *Opa1*^−/−^ (light red squares)). The model, constructed with 31 molecules (shown in **c**), discriminates the *Opa1* genotype on the t([1] + PS[1]) axis. (**c**) VIP plot showing the contribution of each metabolite to the model described in **b**. Molecules emphasized in black (VIP > 1) were considered the most significant molecules of the *Opa1* signature.

To explore the global structure of the data, we first carried out the principal component analysis (PCA) (Fig. 4a). This unsupervised approach highlights any spontaneous clustering or separation of samples according to their global metabolite profile, distributed in the first two most discriminant components (the t[1] and t[2]), explaining the largest variations of the dataset. The PCA model (Fig. 4a) revealed an important difference between the *Opa1*^−/−^ and *Opa1*^+/+^ MEF cell lines with respect to their genotype, delimited by the t[2] axis (R2X[2]: 37.2%). The PCA model showed two clusters within the *Opa1*^+/+^ MEF group, probably representing sub-populations of the controls, whereas the *Opa1*^−/−^ MEFs were well grouped together.

To further the investigation, we used the supervised OPLS-DA (Orthogonal Partial Least Squares-Discriminant Analysis) approach, retaining only the metabolites with the most robust ability of discrimination. This led to the construction of a model (Fig. 4b) with the high predictive ability of 99.8% for Q_2_(cum), well above the threshold value of 0.5 commonly used in metabolomic studies, and with an excellent performance during the permutation test (for 999 permutations, R2: (0.0, 0.0101); Q2: (0.0, −0.471)), and the CV-ANOVA tests (Cross Validation-Analysis Of Variance*, p-*value: 6.45628e^−21^). Moreover, the prediction of the test set samples (n = 6) using this model was correct at 100%, with a Fisher’s combined probability test result of 0.05. Thirty-one metabolites were required to obtain the metabolomic fingerprinting of OPA1 disruption, 14 of which had a VIP (Variable Important for the Projection) score >1, the threshold usually retained in metabolomic studies and considered relevant for the signature (Fig. 4c, in black). Among these 14 molecules, some were directly associated with energetic metabolism (i.e. increase in 1-methylnicotinamide and adenosine 5-monophosphate, and decrease in phosphocreatine), with lipid metabolism pathways (i.e. reduction of sn-glycero-3-phosphocholine phosphocholine), with detoxificationof reactive oxygen species (i.e. a rise in glutathione concentration), with amino acid metabolism (i.e. increase of asparagine and n-acetylputrescine, and decrease of aspartate, pantothenic acid and maleamate), whereas the other molecules could not be properly identified (i.e. a rise concentration of a phenylpropanoids,C_9_H_10_O_4__7.95, an amino acid, C_4_H_9_NO_3__1.02; and a sugar, C_6_H_14_O_6__1.07). Seventeen other metabolites (Fig. 4c, in grey), also important for the signature, but with a VIP < 1, comprised a decrease of cytosine, glutamic acid, cysteic acid, 1-oleyl-rac-glycerol, carnitine and an unnamed amino acid (C_6_H_11_NO_4__1.1), but also an increase of glutamine, proline, serine, glycine, 1-aminocyclopropane1-carboxylate, three other unnamed amino acid (C_3_H_7_NO_2__0.97, C_4_H_9_NO_2__1.06 and C_5_H_9_NO_3__1.02), a lipid not clearly identified (C_7_H_15_NO_2__0.99), uridine-5-monophosphate and n(pai)-methyl-histidine.

Univariate analysis, performed on the 90 accurately measured metabolites, revealed 36 molecules with a fold change (FC) greater than 1.2, and found significant after the Benjamini-Hochberg correction. Fourteen of these had lower concentrations in *Opa1*^−/−^ MEFs than in *Opa1*^+/+^ MEFs, i.e. aspartate, phosphocreatine, phosphocholine, pantothenic acid, sn-glycero-3-phosphocholine, maleamate, cytosine, glutamic acid, C_6_H_11_NO_4__1.1, 1-oleoyl-rac-glycerol, α-ketoglutaric acid, carnitine, creatine, and C_8_H_9_NO_3__1.07) (Table 1). Interestingly, 11 of these 14 metabolites were also found discriminant in the multivariate signature (metabolites shown in bold type), i.e. aspartate, phosphocreatine, phosphocholine, pantothenic acid, sn-glycero-3-phosphocholine, maleamate, cytosine, glutamic acid, C_6_H_11_NO_4__1.1, 1-oleoyl-rac-glycerol, and carnitine), while the remaining three metabolites, i.e. α-ketoglutaric acid, creatine and C_8_H_9_NO_3__1.07, were not present in the OPLS-DA model.

**Table 1:** Listing of the 38 relevant metabolites characterizing the *Opa1*^−/−^ MEF signature.

Modified Metabolites in *Opa1*^−/−^	Family	Univariate			Multivariate
		FC	*p*-value	Corrected threshold	VIP Value (OPLS-DA)
**1-methylnicotinamide**	Vitamin B3	36.2 (↑)	1.08E-05	1.00E-02	2.0
**Asparagine**	Amino acid	10.0 (↑)	1.08E-05	4.44E-03	1.6
**Aspartate**	Amino acid	0.2 (↓)	1.08E-05	3.89E-03	1.3
**Phosphocreatine**	Amino acid derivate	0.2 (↓)	1.08E-05	2.22E-03	1.3
**Phosphocholine**	Lipid precursor	0.3 (↓)	1.08E-05	6.67E-03	1.2
**Pantothenic acid**	Vitamin B5	0.3 (↓)	1.08E-05	1.17E-02	1.2
**Sn-glycero-3-phosphocholine**	Lipid	0.3 (↓)	1.08E-05	1.06E-02	1.2
**Adenosine 5-monophosphate**	Nucleotide	2.9 (↑)	1.08E-05	5.56E-04	1.2
**Maleamate**	Amino acid	0.4 (↓)	1.08E-05	3.33E-03	1.1
**C**_**9**_ **H**_**10**_ **O**_**4**_ **_7.95**	Phenylpropanoic acid	3.2 (↑)	1.08E-05	1.11E-02	1.1
**C**_**4**_ **H**_**9**_ **NO**_**3**_ **_1.02**	Amino acid	2.4 (↑)	1.08E-05	5.56E-03	1.0
**N-acetylputrescine**	Carboximidic acid	2.3 (↑)	1.08E-05	9.44E-03	1.0
**C**_**6**_ **H**_**14**_ **O**_**6**_ **_1.07**	Sugar	2.2 (↑)	4.33E-05	1.33E-02	1.0
**Glutathione**	Tripeptide	1.9 (↑)	1.05E-03	1.67E-02	1.0
**Cytosine**	Nucleoside	0.4 (↓)	1.08E-05	2.78E-03	0.9
**Proline**	Amino acid	2.1 (↑)	1.08E-05	7.22E-03	0.9
**C**_**3**_ **H**_**7**_ **NO**_**2**_ **_0.97**	Amino acid	2.1 (↑)	1.08E-05	1.11E-03	0.9
**Glutamine**	Amino acid	2.0 (↑)	1.08E-05	6.11E-03	0.9
**Serine**	Amino acid	2.0 (↑)	1.08E-05	1.67E-03	0.9
**Glutamic acid**	Amino acid	0.6 (↓)	1.08E-05	8.33E-03	0.8
**C**_**4**_ **H**_**9**_ **NO**_**2**_ **_1.06**	Alpha amino acid	1.9 (↑)	1.08E-05	5.00E-03	0.8
**C**_**5**_ **H**_**9**_ **NO**_**3**_ **_1.02**	Amino acid	1.7 (↑)	1.08E-05	7.78E-03	0.8
**1-oleoyl-rac-glycerol**	Lipid	0.7 (↓)	2.17E-05	1.22E-02	0.7
**C**_**7**_ **H**_**15**_ **NO**_**2**_ **_0.99**	Lipid	1.5 (↑)	3.25E-04	1.56E-02	0.7
Cysteic acid	Amino acid	1.4 (↑)	NS		0.7
**C**_**6**_ **H**_**11**_ **NO**_**4**_ **_1.1**	Alpha amino acid	0.7 (↓)	1.08E-05	8.89E-03	0.6
**Carnitine**	Carnitine	0.7 (↓)	7.25E-04	1.61E-02	0.6
**1-Aminocyclopropane-1-carboxylate**	Alpha amino acid	1.3 (↑)	2.17E-05	1.28E-02	0.5
**Glycine**	Amino acid	1.3 (↑)	3.25E-04	1.44E-02	0.5
**Uridine-5-monophosphate**	Nucleotide	1.2 (↑)	5.20E-03	1.89E-02	0.4
N(PAI)-methyl-histidine	Carboxylic acid	<1.2 (↑)	1.47E-02	2.11E-02	0.4
α-ketoglutaric acid	Keto acid	0.6 (↓)	7.58E-05	1.39E-02	
Creatine	Amino acid derivate	0.7 (↓)	3.89E-03	1.78E-02	
Adenine	Purine	1.9 (↑)	5.78E-03	1.94E-02	
Hypotaurine	Sulfinic acid	1.31 (↑)	3.25E-04	1.50E-02	
C_8_H_9_NO_3__1.07	Alpha amino acid	0.7 (↓)	3.89E-03	1.83E-02	
Choline	Vitamin	1.4 (↑)	2.88E-03	1.72E-02	
Pyruvate	Keto acid	1.7 (↑)	1.15E-02	2.06E-02	

Molecules were sorted by decreasing VIP values. For each metabolite, the table shows the FC (fold change), considered as the ratio between the mean values of the two groups, *Opa1*^−/−^ *vs. Opa1*^+/+^ MEF cell lines; and the *p*-values obtained in the univariate analysis after application of the Wilcoxon test with the new threshold of significance obtained with the Benjamini-Hochberg correction. Molecules common to the signatures found with the multivariate and the univariate analyses are shown in bold type. NS: not significant (*p-*value: 0.10512).

The 22 other metabolites (Table 1) had higher concentrations in *Opa1*^−/−^ MEFs than in *Opa1*^+/+^ MEFs, i.e. 1-methylnicotinamide, asparagine, C_9_H_10_O_4__7.95, adenosine 5-monophosphate, C_4_H_9_NO_3__1.02, n-acetylputrescine, proline, C_3_H_7_NO_2__0.97, glutamine, serine, C_4_H_9_NO_2__1.06, C_5_H_9_NO_3__1.02, 1-aminocyclopropane-1-carboxylate, C_6_H_14_O_6__1.07, C_7_H_15_NO_2__0.99, glycine, glutathione, uridine-5-monophosphate, adenine, pyruvate, hypotaurine, and choline. Among these, 18 were common to the OPLS-DA signature (compounds shown in bold type), i.e. 1-methylnicotinamide, asparagine, C_9_H_10_O_4__7.95, adenosine 5-monophosphate, C_4_H_9_NO_3__1.02, n-acetylputrescine, proline, C_3_H_7_NO_2__0.97, glutamine, serine, C_4_H_9_NO_2__1.06, C_5_H_9_NO_3__1.02, 1-aminocyclopropane-1-carboxylate, C_6_H_14_O_6__1.07, C_7_H_15_NO_2__0.99, glycine, glutathione, and uridine-5-monophosphate. Finally, four novel significant metabolites, i.e. adenine, pyruvate, hypotaurine, and choline, were identified by the univariate analysis, although absent in the OPLS-DA model.

The combination of multivariate and univariate analyses revealed 29 common discriminant metabolites (shown in bold type in Table 1), i.e. a decrease of aspartate, phosphocreatine, phosphocholine, pantothenic acid, sn-glycero-3-phosphocholine, maleamate, cytosine, glutamic acid, C_6_H_11_NO_4__1.1, 1-oleoyl-rac-glycerol and carnitine, and an increase of 1-methylnicotinamide, asparagine, C_9_H_10_O_4__7.95, adenosine 5-monophosphate, C_4_H_9_NO_3__1.02, n-acetylputrescine, proline, C_3_H_7_NO_2__0.97, glutamine, serine, C_4_H_9_NO_2__1.06, C_5_H_9_NO_3__1.02, 1-aminocyclopropane-1-carboxylate, C_6_H_14_O_6__1.07, C_7_H_15_NO_2__0.99, glycine, glutathione in reduced form and uridine-5-monophosphate. Among these 29 metabolites, 14 were particularly relevant not only because of the VIP score >1 but also because of the high FC > 1.5, i.e. higher concentration in 1-methylnicotinamide, asparagine, adenosine 5-monophosphate, C_9_H_10_O_4__7.95, C_4_H_9_NO_3__1.02, n-acetylputrescine, C_6_H_14_O_6__1.07 and glutathione and reduce level of maleamate, aspartate, phosphocreatine, phosphocholine, pantothenic acid and sn-glycero-3-phosphocholine.

### Aspartate supplementation

**Figure 5:**
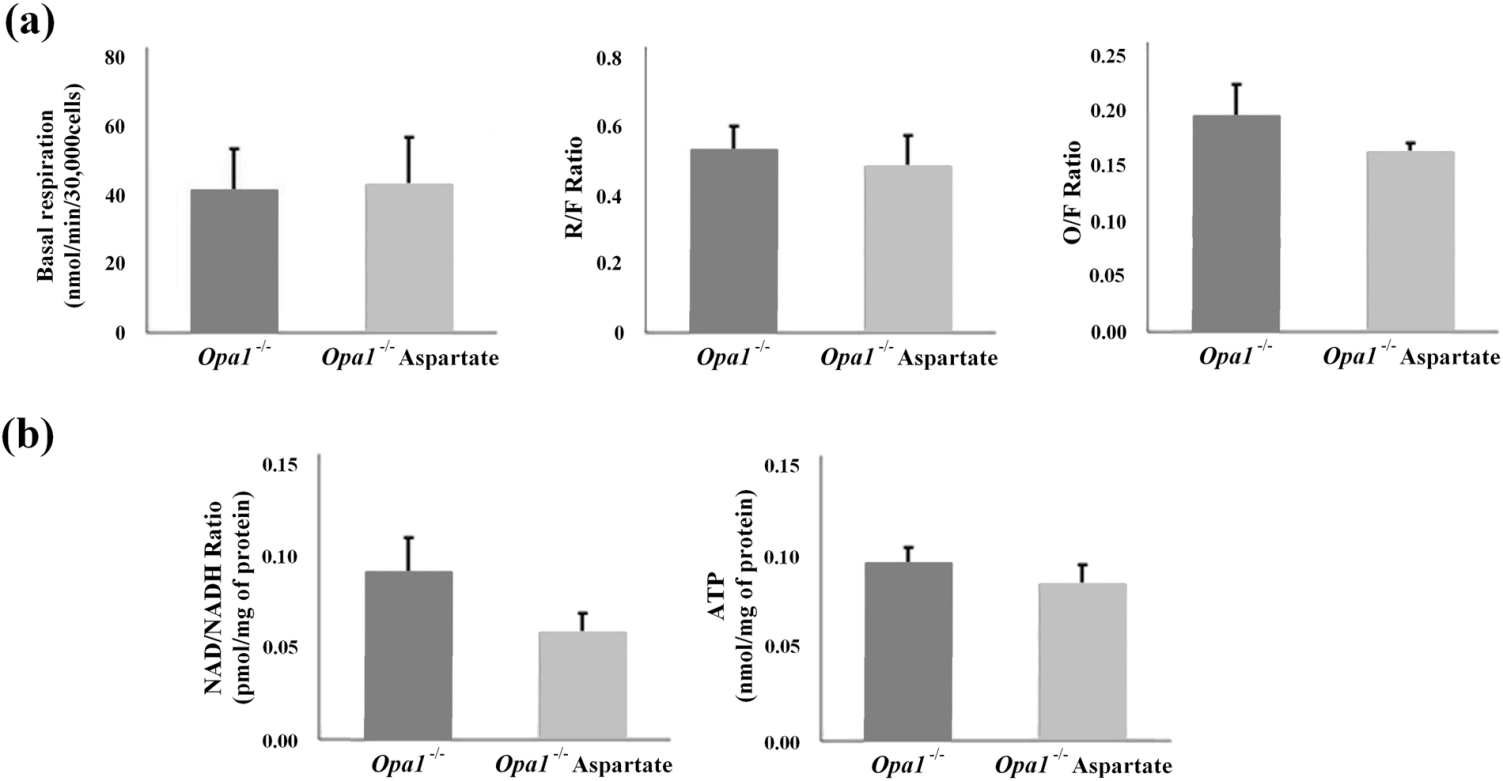
Aspartate supplementation. (**a**) Oxygen consumption rates were measured in *Opa1*^−/−^ MEFs either treated or not with 20 mM aspartate for 48 h with the Seahorse XFe96 extracellular flux analyser. OCR was evaluated with the following injection protocol: oligomycin (2 μg/mL), FCCP1 (0.25 μM) and FCCP2 (1.5 μM in this case) and antimycin A (2 μg/mL). Histograms show the mean ± S.D. of four independent experiments. (**b**) NAD/NADH ratio and ATP in *Opa1*^−/−^ MEFs incubated in glucose medium supplemented with 20 mM aspartate for 48 h. Histograms show the mean ± S.D. of three independent experiments for the intracellular NAD/NADH and five independent experiments for the cellular ATP content. NAD, NADH and ATP values were normalised by protein concentration. The statistical analysis was carried out using Student’s paired *t‐*test.

Overall, the data points to a severe aspartate deficiency in the *Opa1*^−/−^ MEFs, thus we sought to evaluate whether aspartate supplementation may rescue mitochondrial function. We measured parameters of mitochondrial function including mitochondrial respiration, NAD/NADH ratio and cellular ATP content following aspartate supplementation. *Opa1*^−/−^ cells showed similar levels of basal respiration and other components of the bioenergetics function, including the respiratory capacity (R/F) and the non-phosphorylating respiration (O/F) after 48 h of aspartate treatment (Fig. 5a). In addition, aspartate did not modified the NAD/NADH ratio in *Opa1*^−/−^ cells compared to their untreated counterparts (respectively, 0.05 (±0.010) vs 0.09 (±0.019)) (Fig. 5b). Cellular ATP content remained unchanged in untreated *Opa1*^−/−^ (0.097 (±0.008) compared with *Opa1*^−/−^ treated with 20 mM aspartate for 48 h (0.0857 (±0.0107)) (Fig. 5b).

## Discussion

In this study, we aimed at obtaining a metabolomic overview of complete *Opa1* disruption. Among the 500 metabolites screened by our non-targeted metabolomic approach, 90 were accurately detected, and variations of the concentrations of 38 of these were found relevant for differentiating between the *Opa1*^−/−^ and the *Opa1*^+/+^ MEF cell lines (Table 1). Univariate analysis highlighted 36 metabolites whereas our multivariate model identified 31 metabolites, with 29 of these being common to the two statistical approaches used. Moreover, with the most stringent statistical criteria, VIP > 1 and FC > 1.5, 14 metabolites turned out to be the most discriminant molecules.

**Figure 6:**
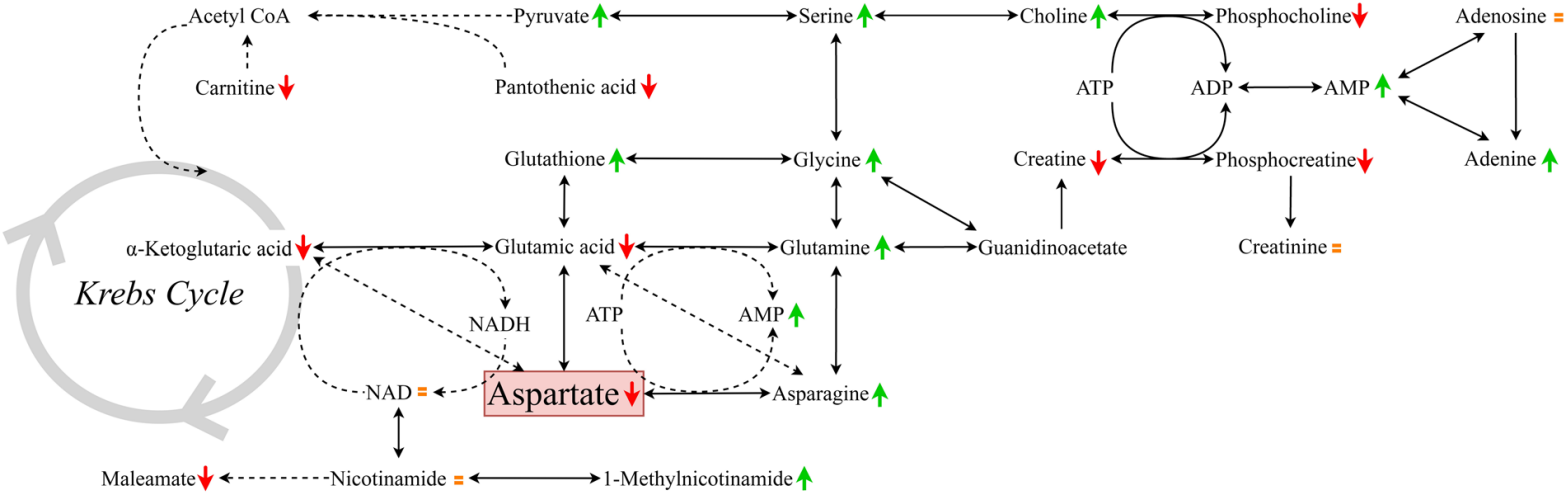
Model summarizing the changes in metabolite concentrations in *Opa1*^−/−^ MEFs compared to *Opa1*^+/+^ MEFs. The pathways show increased (green arrows), decreased (red arrows) or unchanged (orange ‘=’ sign) molecular concentrations in the *Opa1*^−/−^ cell line after the statistical analyses.

Although the metabolome of the entire cells was studied, the majority of discriminant metabolites identified were involved in mitochondrial metabolism, suggesting the presence of an extensive, closely coordinated metabolic network (Fig. 6). This network comprises a central group of seven altered, tightly inter-connected metabolites, i.e. aspartate (FC = 0.2), asparagine (FC = 10.0), glutamine (FC = 2.0), glutamic acid (FC = 0.6), α-ketoglutaric acid (FC = 0.6), 1-methylnicotinamide (FC = 36.2) and adenosine-monophosphate (FC = 2.9), in *Opa1*^−/−^ MEFs compared to *Opa1*^+/+^ MEFs. All these molecules are implicated in the aspartate/malate shuttle, which ensures the equilibrium of the NAD/NADH ratio on each side of the mitochondrial inner membrane. As shown in Fig. 2, mitochondrial respiration is profoundly affected by the disruption of OPA1, thus altering the ability for re-oxidizing the NADH and modifying the NAD/NADH ratio. The increased concentration of 1-methylnicotinamide, which is a direct metabolite of NAD, is probably a sign of impaired NAD metabolism. Maleamate (FC = 0.4) may also be involved in the metabolism of nicotinamide^27^, but its precise role is poorly understood. Since NAD is a direct cofactor of the cytosolic and mitochondrial isoforms of malate dehydrogenase in this shuttle, the decreased concentration of glutamate, α-ketoglutaric acid, and aspartate is metabolically consistent with the reduced NAD/NADH ratio. In addition, the transamination of aspartate to asparagine in parallel to the deamination of glutamine to glutamate, which requires ATP hydrolysis, is also profoundly affected, reinforcing the notion of the central involvement of this aspartate-related metabolic pathway.

The reduced aspartate level found in the *Opa1*^−/−^ MEFs compared to *Opa1*^+/+^ MEFs particularly drew our attention for three reasons. Firstly, we had recently reported a plasma metabolomic signature characterized by altered purine metabolism in patients with various phenotypes caused by pathogenic variants of *OPA1*^24^. Although there were several metabolite variations similar to those in the present *Opa1*^−/−^ MEF profile, i.e. decrease of aspartate, glutamate and 1-oleoyl-rac-glycerol, other metabolites in human plasma, displayed opposite variations, i.e. reduction choline and increase of phosphocholine. The lower concentrations of aspartate and glutamate may be particularly relevant, since they were found in the patients’ blood as well as in the *Opa1*^−/−^ MEF model.

Secondly, two recent articles have highlighted the primary role of the respiratory chain in the production of aspartate, a limiting metabolite for the proliferation of cells with respiratory chain inhibition^28,29^. The disruption of the respiratory chain was shown to reduce aspartate synthesis and cell proliferation, whereas aspartate supplementation supported cell proliferation even in the absence of a functional respiratory chain. Our results, supporting pathophysiological evidence that aspartate deficiency results from respiratory chain disruption, also provide a larger overview of the metabolic changes accompanying aspartate deficiency. In addition, aspartate was found to be the most abundant polar metabolite among the 132 metabolites recently quantified by metabolomics in purified mitochondria^30^. However, the blood concentration of aspartate being physiologically very low^28^, it is unlikely that cells *in vivo* with respiratory chain deficiencies could import sufficient quantities of aspartate to compensate for any altered biosynthesis.

Thirdly, aspartate has been shown to be necessary for the *de novo* synthesis of purine and pyrimidine^29^, and we have previously reported the sharp alteration of purine metabolism in the plasma of individuals carrying an *OPA1* mutation^24^. Similarly, our present study on *Opa1*^−/−^ MEFs reveals an adaptation of the nucleotide metabolism, with cytosine (FC = 0.4) and AMP (FC = 2.9), which might be partially related to impaired OXPHOS (OXidative PHOSphorylation) activity, with uridine-5′-monophosphate (FC = 1.2) and adenine (FC = 1.9). The interactions between respiratory chain defects, aspartate deficiency and altered nucleotide metabolism may therefore be relevant to the pathophysiology of OPA1-related disorders.

Based on the above aspartate dysfunction and its functional consequences, aspartate supplementation arose as a therapeutic alternative in *OPA1* dysfunctions. Unfortunately, levels of aspartate comparable to the ones used in Sullivan *et al*. study failed to restore mitochondrial respiration in MEFs *Opa1*^−/−^ cells. These results are consistent with the previous findings of Sullivan *et al*. which showed that had no impact on NAD/NADH ratio and mitochondrial oxygen consumption^29^.

Concerning the variation of the other metabolites involved in the disruption of OPA1 in MEF cell lines, it is not surprising to find components of energetic metabolism such as phosphocreatine (FC = 0.2) and creatine (FC = 0.7), which are both involved in the buffering of ATP and the shuttling of energy into the cytosol, as well as pyruvate (FC = 1.7), which may accumulate in mitochondria as a consequence of the decreased oxidation of the energetic substrate. Pantothenic acid (FC = 0.3) is a component of acetyl-CoA, which is the main substrate of the tricarboxylic acid cycle. Carnitine (FC = 0.7) is also a major cofactor of the energetic metabolism through its role in fatty acid transport across the mitochondrial inner membrane. The increased concentration of glutathione (FC = 1.9) also attests to the presence of oxidative stress due to respiratory chain defects.

The increased levels of serine (FC = 2.0) and choline (FC = 1.4) together with the decreased levels of phosphocholine (FC = 0.3) and sn-glycero-3-phosphocholine (FC = 0.3) are also consistent with impaired ATP production, since serine and choline are substrates for the synthesis of ATP-dependent phosphocholine, which is a precursor of phosphatidylcholines, the most abundant membrane phospholipids. The variations of the remaining metabolites, i.e. proline, 1-oleoyl-rac-glycerol, N-acetylputrescine, cysteic acid, glycine and methyl-histidine, are more difficult to interpret in the context of the disruption of OPA1 and the respiratory chain.

In conclusion, the disruption of OPA1 in MEF cell lines leads to a significant metabolomic signature, emphasizing a profound, pleiotropic impairment of the energetic metabolism, downstream with respect to the OXPHOS deficiency. The aspartate defect, at the heart of this signature, and which is also present in the plasma of individuals affected with DOA caused by pathogenic *OPA1* variants, seems to play a central role in the pathophysiology of the disease.

## Methods

### Chemicals and reagents

Methanol (MeOH), water, formic acid (Optima LC/MS grade) and glutamine were purchased from Thermo-Fisher Scientific (Illkirch, France). Isotope metabolite standards including 17α-Hydroxyprogesterone-d_8_ (2,2,4,6,6,21,21,21-d8), L-Thyroxine-^13^C_6_, Succinic acid-2,2,3,3-d_4_, Pyruvic acid-1-^13^C and DL-Alanine-^15^N with >98% purity were acquired from Sigma Aldrich (St. Quentin Fallavier, France) as well as oligomycin, carbonyl cyanide 4‐(trifluoromethoxy) phenylhydrazone (FCCP), antimycin A and aspartate. All antibodies (ab42364, EP1332Y, ab186695 and ab186696), the NAD/NADH and ATP Assay Kit (ab65348 and ab83355) were obtained from Abcam (Paris, France) and the Mitotracker® green from Molecular Probes (Oregon, USA). Tris-Glycine Gel was purchased from Life Technologies (Illkirch, France) and DMEM-F12 from Jacques Boy Institute of Biotechnology (Reims, France). The DMEM medium supplemented with FBS (fetal bovine serum) was acquired from PAN-biotech (Wimborne, UK) and the Seahorse XFe Base Medium from Agilent Technologies (Santa Clara, CA, USA).

### Cell cultures

Immortalized mouse embryonic fibroblasts (MEFs) from *Opa1*^−/−^ knockout C57BL/6 mice and *Opa1*^+/+^ wild-type controls were cultivated in a medium consisting of Dulbecco’s modified Eagle medium with nutrient mixture F12 (DMEM-F12) supplemented with 10% FBS at 37 °C, 5% CO_2_. Both cell lines shared the same passage numbers. The analyses of *Opa1*^−/−^ and *Opa1*^+/+^ MEF cell lines were all performed within the same exponential growth stage. When needed, cell culture medium was supplemented with 20 mM aspartate at pH 7.4 (±0.2) for 48 h.

### Western blot analysis

Eight million cells of each MEF cell line were collected before confluence (n: 5). Ice-cold radio-immunoprecipitation assay (RIPA) buffer was utilized to lyse samples for 15 min at 4 °C followed by centrifugation at 20,000 g at 4 °C for 20 min. Protein extracts (30 μg) were separated on 4–20% Tris-Glycine Gel and electron-transferred to nitrocellulose membranes according to standard procedures. After blocking the free binding sites with 5% BSA (bovine serum albumin) reconstituted in phosphate-buffered saline with 0.2% Tween-20, the membranes were probed with anti-Opa1 (ab42364) and anti-tubulin-α (EP1332Y) antibodies. Anti-mouse and anti-rabbit fluorescence (1:10,000) (ab186695 and ab186696, respectively) were used as secondary antibodies. The images were quantitatively acquired using Image Studio 2.1 software (Li-Cor, Lincoln, NE, USA).

### Mitochondrial oxygen consumption

Cells were seeded in XF Cell Culture Microplates (Seahorse, Agilent Technologies, Santa Clara, CA, USA) at a concentration of 30,000 cells/well in 100 μL DMEM 4.5 g/L medium supplemented with 10% FBS and 1 mM glutamine and incubated for 6 h at 37 °C in 5% CO_2_ atmosphere. Before the experiments, the culture medium was removed from each well and replaced by 100 μl of Seahorse XFe Base Medium pre-warmed at 37 °C and supplemented with 1 mM glutamine, pH 7.4 (±0.4). Cells were incubated in a CO_2_-free incubator at 37 °C for 1 h. Before the measurements, the XFe Extracellular Flux Analyzer (Seahorse, Agilent Technologies, Santa Clara, CA, USA) automatically mixed the assay media in each well for 10 min to allow the oxygen partial pressure to reach equilibrium and three baseline measurements were taken before the addition of any of the compounds loaded in the cartridge. Injection ports on the sensor cartridge were loaded with oligomycin (2 μg/mL), a titration of carbonyl cyanide 4‐ (trifluoromethoxy) phenylhydrazone (FCCP) ranging from 0.25 μM to 3 μM, and antimycin A (2 μg/mL). OCR (oxygen consumption rate) values refer to the average oxygen consumption rates during the measurement cycles, which in this case consisted of a 3 min wait, 3 response measurements before and after the addition of the compounds. OCR values were normalised to the number of cells/well.

### NAD/NADH and ATP measurement

Total cellular NAD, NADH and ATP was carried out using the NAD/NADH and ATP assay kit according to the manufacturer’s instructions.

### 3D fluorescence imaging

Cells were seeded on coverslips in AQ4 DMEM-F12, 1% FCS, in a humidified atmosphere (95% air, 5% CO_2_) at 37 °C. Cells were then incubated for 15 min with 100 nM Mitotracker® green to stain the mitochondrial network. Images were acquired with an inverted wide-field microscope ECLIPSE Ti-E (Nikon, Amsterdam, Netherlands) equipped with a 100x oil-immersion objective (Nikon Plan Apo100x, N.A. 1.45) and an Andor NEO sCOMS camera controlled by Metamorph® 7.7 software (Molecular Devices, Sunnyvale, CA, USA). Twenty-one image planes were acquired along the Z-axis at 0.2 μm increments. Following image acquisition, images were first iteratively deconvolved using Huygens Essential® software (Scientific Volume Imaging, Hilversum, The Netherlands), with the maximum iteration scored at 50 and a quality threshold at 0.01, followed by 3D processing and morphometric analysis with Imaris 8.0® software (Bitplane, Zurich, Switzerland). Thirty cells of each MEF cell line were analysed for the quantitative analysis of mitochondrial shapes.

### Metabolomics analysis

Samples (n = 10 for each MEF cell line) were randomly prepared as follows. After removal of the medium, the cellular monolayer was rinsed twice with an aqueous solution containing 0.22% NaCl before being quenched with cold MeOH. The cell suspension (estimated at 4 million cells), obtained after mechanical scraping, was then collected and stored at −80 °C until analysis in an aliquot of 10^6^ cells. Test samples of each cell line (n = 3 samples/cell line) were pooled from various aliquots to validate the statistical model. Internal quality controls (QCs) were generated by mixing all the samples together.

A non-targeted reverse phase (RP) metabolomics method was validated for cell culture samples (Supplementary Table S2). Briefly, a mixture of H_2_O/MeOH was added to 1 million cells, which were initially fortified with the isotope metabolite standards mixture (10 μg/mL in MeOH), in order to achieve a final volume of 600 µL containing 20% water and 80% MeOH. After centrifugation, supernatants were evaporated to dryness. Samples were then reconstituted with an aqueous solution (2% MeOH) before the UHPLC-HRMS (Ultra High Pressure Liquid Chromatography-High Resolution Mass Spectrometry) analysis.

A Thermo Scientific Q Exactive mass spectrometer (Thermo Fisher Scientific, Bremen, Germany), equipped with a heated electrospray ionization source, was used for this study in positive and negative modes. Ionisation conditions and MS parameters were identical to those previously described^23^. Chromatography was carried out using a Dionex UltiMate® 3000 UHPLC (Dionex, Sunnyvale, CA, U.S.A.) equipped with a Phenomenex Kinetex 1.7 µm XB-C18 (150 mm × 2.10 mm, 100 Å) UHPLC column kept at 40 °C. A multi-step gradient (preceded by an equilibration time of about 3 minutes), with an aqueous mobile phase A with 0.1% formic acid and a methanolic mobile phase B with 0.1% of formic acid, was employed with a flow rate maintained at 0.3 ml/min during a total runtime of 22.5 min. The UHPLC autosampler temperature was set at 4 °C.

Metabolite identification was facilitated using the MSMLS^TM^ (Mass Spectrometry Metabolite Library of Standards) molecule library (IROA Technologies, Bolton, MA, U.S.A.) for mass spectrometry metabolomics. These commercial standards were injected in the same analytical conditions in order to create an in-house database (a final collection of 500 accurately identified carboxylic acids, amino acids, nucleotides, saccharides, fatty acids, lipids and hormones). Then, a TraceFinder 4.1 processing method was designed to allow a comparison of the Retention Times (RT), the MS/MS fragmentation spectra, the m/z and the isotopic patterns of metabolites. Ions integrated with a CV (Coefficient of Variation) higher than 30% in QC, a RT drift greater than 10 sec, and a linearity of dilution with an r² lower than 0.7 were immediately discarded. The identification criteria included accurate measurement of the m/z ratio (better than 5 ppm), a perfect isotopic pattern, an RT drift lesser than 5 s, and/or the presence of two identical fragments (level of metabolite identifications: 1, identified compounds)^31^. Otherwise, molecules were named by their chemical formula and RT (level of metabolite identifications: 2, putatively annotated compounds)^31^.

### Statistical analyses

Before performing statistical analyses, data were normalised by the total ion current (TIC) of each sample using Microsoft Excel software. In addition, the dataset was *log*_10_ transformed, mean-centred and scaled by the square root of the standard deviation of each variable (Pareto scaling) to reduce the contribution of the most intense ions. Statistical analyses were performed following the workflow chart shown in Fig. 3.

Multivariate analysis was carried out with Simca-P + v 14.0 (Umetrics, Umea, Sweden). Principal Component Analysis (PCA), an unsupervised method, was used to investigate the population structure and to emphasize spontaneous clustering or separation of samples on the basis of their global metabolite profiles. To highlight molecules implicated in the metabolomic signature, Orthogonal Partial Least Squares Discriminant Analysis (OPLS-DA), a supervised analysis, was carried out, retaining only the metabolites that showed a strong power of discrimination and a high statistical reliability in the model. This means that variables were gradually excluded according to the results obtained from the different plots, i.e. the S-plot (visualization of intensity and reliability), the loading column plot with jack-knife confidence intervals, the coefficient plot, and finally, the Variable Importance in the Projection (VIP) plot. The purpose was to minimize the risk of over-fitting and to reduce the variability of prediction, thus simplifying interpretation of the molecular signature. OPLS-DA models were cross-validated by leaving out one-third of the samples, and this process was replicated three times. The qualities and performances of the models were evaluated using the Q²Ycum (goodness of prediction), the R²Ycum (goodness of fit) values, the cross validation-analysis of variance (CV-ANOVA), the permutation test (evaluation of the risk of over-fitting) and the prediction of a test set (6 test samples prepared and analysed like the other samples but not used for the development of the statistical model). Finally, only metabolites with a VIP value greater than 1 were considered as “highly relevant” for the metabolomic foot-printing.

Univariate analysis was performed with MetaboAnalyst 3.5^32^ using the Volcano plot module. Consequently, only metabolites with an FC greater than 1.2 and a Wilcoxon test at a 0.05 threshold were considered. The Benjamini-Hochberg correction was applied to minimize the error rate, and only the molecules that remained significant were retained.

Statistical analysis of the western blots, mitochondrial respiration, and quantitative mitochondrial imaging was done with GraphPad statistical software (San Diego, CA, USA). The non-parametric Mann-Whitney test was used for western blot analysis and Student’s unpaired *t-*test was used for imaging and respiration analysis. For all analyses, a *p-*value < 0.05 was considered statistically significant.

## Electronic supplementary material

Supplementary file
