# Supplementary material for: The Metabolomic Bioenergetic Signature of Opa1-Disrupted Mouse Embryonic Fibroblasts Highlights Aspartate Deficiency
Source: Sci Rep. 2018 Aug 1;8:11528. doi: 10.1038/s41598-018-29972-9 (PMC6070520; doi:10.1038/s41598-018-29972-9)
Supplement: Supplementary file 1 — Supplementary file [file 41598_2018_29972_MOESM1_ESM.pdf]

THE METABOLOMIC BIOENERGETIC SIGNATURE OF *Opa1*-DISRUPTED MOUSE EMBRYONIC FIBROBLASTS HIGHLIGHTS ASPARTATE DEFICIENCY

Cinzia Bocca, MSc,[1] Mariame-Selma Kane, PhD,[1] Charlotte Veyrat-Durebex, PharmD, PhD,[1,2] Stéphanie Chupin, Tech,[2] Jennifer Alban, Tech,[1] Judith Kouassi Nzoughet, PhD,[1] Morgane Le Mao, MSc,[1] Juan Manuel Chao de la Barca, MD, PhD,[1,2 3] Patrizia Amati-Bonneau, MD,[1,2] Dominique Bonneau, MD, PhD,[1,2] Vincent Procaccio, MD, PhD,[1,2] Guy Lenaers, PhD,[1] Gilles Simard, MD, PhD,[2,4] Arnaud Chevrollier, PhD,[1] Pascal Reynier, MD, PhD[1,2*]

[1]Equipe Mitolab, Institut MITOVASC, CNRS 6015, INSERM U1083, Université d'Angers, Angers, France

[2]Département de Biochimie et Génétique, Centre Hospitalier Universitaire, Angers, France

[3]CNRS, Institut de Biochimie et Génétique Cellulaires, UMR 5095, Bordeaux, France.

[3]INSERM U1063, Université d’Angers, Angers, France

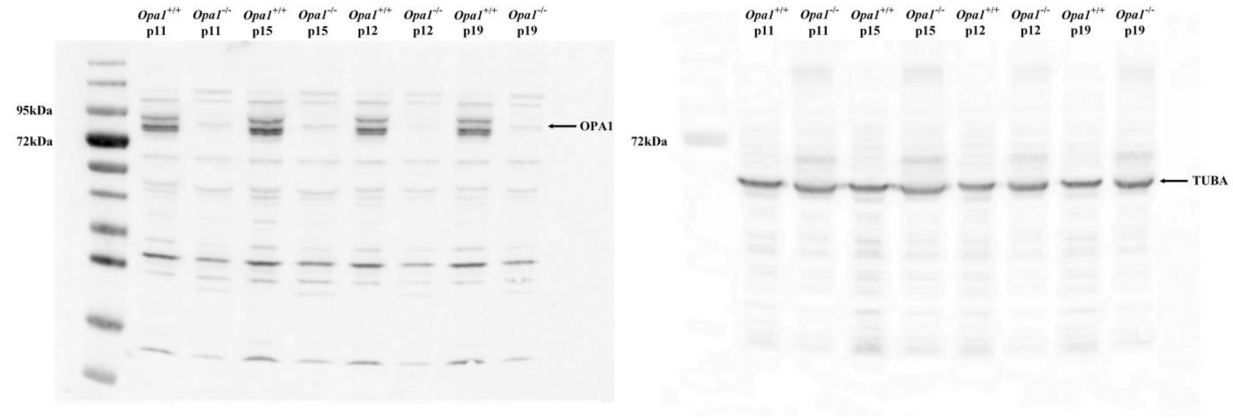

Supplementary Figure S1: **Full-length blots of OPA1 protein expression.** Thirty µg of protein from cell lysates were loaded on the gel and revealed after transfer by OPA1 and TUBA antibodies. Four different passages (p11-p19) of culture amplification were done for each cell line.

1-aminocyclopropane-1-carboxylate	$C_6H_{11}NO_4$_1.22	Leucine
1-methylnicotinamide	$C_6H_{12}O_5$_1.2	Malate
1-oleoyl-rac-glycerol	$C_6H_{12}O_6$_1.07	Maleamate
3-sulfino-l-alanine	$C_6H_{12}O_7$_1.2	Methionine
5-hydroxy-tryptophan	$C_6H_{13}O_9P$_1.5	N(Pai)-methyl-histidine
5-methylthioadenosine	$C_6H_{14}O_6$_1.07	N-acetylneuraminate
5-oxo-proline	$C_6H_5NO_2$_1.51	N-acetylputrescine
Adenine	$C_7H_{15}NO_2$_0.99	N-acetylserotonin
Adenosine	$C_8H_9NO_3$_1.07	NAD
Adenosine-5-monophosphate	$C_9H_{10}O_4$_7.95	Nicotinamide
Arginine	Carnitine	Pantothenic acid
Asparagine	Choline	Phenylalanine
Aspartate	CMP	Phosphocholine
$C_{12}H_{22}O_{11}$_1.1	Creatine	Phosphocreatine
$C_{15}H_{24}N_2O_{17}P_2$_6.5	Creatinine	Pipecolate
$C_{17}H_{27}N_3O_{17}P_2$_7.3	Cysteic acid	Proline
$C_3H_4O_3$_1.1	Cytosine	Pyridoxine
$C_3H_7NO_2$_0.97	Dihydroorotate	Pyruvate
$C_3H_9O_6P$_1.5	D-ribose 5-phosphate	S-(5-adenosyl)-methionine
$C_4H_4O_4$_1.98	Folic acid	Serine
$C_4H_6O_3$_1.85	Glutamic acid	SN-glycero-3-phosphocholine
$C_4H_9NO_2$_1.06	Glutamine	Stearate
$C_4H_9NO_3$_1.02	Glutathione	Succinate
$C_5H_{10}O_5$_1.1	Glyceraldehyde	Taurine
$C_5H_{12}O_5$_1.06	Glycine	Thiamine
$C_5H_7NO_3$_1	Histidine	Tryptophan
$C_5H_9NO_3$_1.02	Hypotaurine	Tyrosine
$C_6H_{10}O_3$_12.2	Isoleucine	Uridine-5-monophosphate
$C_6H_{10}O_3$_12.9	Kynurenine	Valine
$C_6H_{11}NO_4$_1.1	Lactate	α-ketoglutaric acid

Supplementary Table S1: The 90 metabolites detected and quantified from positive and negative ionisation modes

	Within-day CV (%)			Between-day CV (%)	Within−day precision CV (%)	Between−day precision CV (%)	Intermediate precision CV (%)
	**Day 01**	**Day 02**	**Day 03**				
Lactate	1.41%	2.39%	2.13%	2.22%	2.03%	1.08%	2.30%
1-aminocyclopropane-1-carboxylate	2.52%	2.13%	1.34%	2.07%	2.05%	0.33%	2.08%
1-methylnicotinamide	2.80%	2.67%	1.56%	3.02%	2.42%	2.18%	3.26%
Amino-methylpropanoate	5.10%	3.97%	2.46%	4.39%	3.95%	2.31%	4.58%
3-ureidopropionate	3.93%	2.39%	3.00%	3.12%	3.17%	0.65%	3.10%
5,6-dihydrouracil	3.81%	2.50%	3.87%	3.38%	3.46%	0.88%	3.34%
5-oxo-proline	1.10%	1.48%	0.99%	1.19%	1.21%	0.24%	1.18%
Adenine	12.93%	8.49%	9.06%	12.21%	10.09%	8.30%	13.06%
Adenosine	10.72%	5.15%	2.88%	7.84%	6.79%	4.74%	8.28%
Allantoin	1.23%	3.06%	2.15%	2.39%	2.26%	0.94%	2.45%
A-KETOGLUTARIC ACID	1.88%	3.56%	2.86%	3.24%	2.87%	1.83%	3.40%
Aspartate	2.60%	2.00%	2.05%	2.24%	2.24%	0.11%	2.24%
Choline	2.70%	1.22%	0.91%	1.98%	1.81%	0.99%	2.06%
Citramalate	4.04%	4.76%	3.15%	3.95%	4.05%	1.04%	3.91%
Citrate	7.13%	6.72%	5.18%	7.03%	6.38%	3.56%	7.30%
Cmp	4.42%	3.41%	3.31%	3.77%	3.75%	0.45%	3.78%
Creatine	3.98%	1.18%	0.70%	2.76%	2.39%	1.68%	2.92%
Creatinine	3.10%	3.60%	2.27%	3.07%	3.03%	0.56%	3.09%
Cytidine diphosphocholine	7.62%	6.30%	6.48%	6.74%	6.81%	1.21%	6.71%
Cytosine	13.21%	4.73%	4.94%	9.69%	8.94%	4.51%	10.02%
Docosahexaenoic acid	22.50%	10.53%	5.51%	17.95%	15.97%	9.90%	18.79%
Pantothenic acid	3.41%	3.53%	2.45%	3.11%	3.17%	0.75%	3.08%
Ribose phosphate	5.88%	6.47%	7.45%	7.03%	6.66%	2.75%	7.20%
Folic acid	6.46%	4.99%	4.25%	5.35%	5.34%	0.41%	5.35%
Glutathione	8.83%	10.43%	2.61%	8.83%	8.10%	4.24%	9.14%
Glyceraldehyde	2.58%	2.59%	1.70%	2.29%	2.32%	0.46%	2.28%
Glycerate	1.29%	1.65%	1.87%	1.71%	1.62%	0.66%	1.75%
Glycine	6.17%	6.09%	4.27%	5.48%	5.56%	1.16%	5.44%
Guanosine	3.35%	3.41%	2.83%	3.17%	3.21%	0.59%	3.15%
Hippurate	3.37%	3.68%	2.91%	3.28%	3.34%	0.76%	3.25%
Hypotaurine	2.51%	4.78%	3.47%	3.77%	3.68%	0.95%	3.80%
Hypoxanthine	11.18%	3.49%	1.99%	8.68%	7.26%	5.74%	9.26%
Inosine	2.44%	3.33%	2.69%	2.83%	2.85%	0.37%	2.82%
Kynurenine	2.84%	3.61%	2.97%	3.42%	3.16%	1.56%	3.53%
Arginine	1.91%	1.81%	1.28%	1.67%	1.69%	0.31%	1.66%
Asparagine	3.72%	6.75%	4.93%	5.24%	5.25%	0.40%	5.24%
Carnitine	1.98%	5.80%	3.86%	4.26%	4.14%	1.21%	4.32%
Cysteic acid	2.20%	4.00%	4.46%	3.61%	3.68%	0.86%	3.58%
Cysteine	9.44%	26.83%	8.82%	16.88%	17.09%	3.25%	16.78%
Glutamic acid	3.66%	2.40%	1.49%	2.74%	2.66%	0.83%	2.78%
Glutamine	1.96%	4.38%	3.15%	3.47%	3.29%	1.34%	3.55%
Histidine	3.10%	1.44%	1.10%	2.09%	2.08%	0.23%	2.09%
Isoleucine	2.43%	6.40%	5.01%	5.54%	4.97%	2.95%	5.78%
Phenylalanine	1.14%	3.47%	2.54%	2.76%	2.59%	1.15%	2.83%
Proline	2.71%	1.05%	1.11%	2.25%	1.77%	1.66%	2.43%
Serine	4.20%	5.24%	3.94%	4.39%	4.49%	1.13%	4.35%
Tyrosine	4.93%	3.70%	3.83%	4.09%	4.19%	1.11%	4.04%
Malate	8.67%	6.60%	4.77%	7.68%	6.77%	4.38%	8.06%
Maleamate	3.82%	4.34%	2.77%	3.66%	3.69%	0.61%	3.64%
N(pai)-methyl-l-histidine	6.11%	7.90%	3.59%	5.98%	6.10%	1.44%	5.93%
N-acetyl-glutamic acid	6.65%	6.99%	3.95%	6.83%	6.02%	3.90%	7.18%
N-acetyl-alanine	2.20%	4.18%	3.01%	3.44%	3.25%	1.34%	3.52%
N-acetylneuraminate	5.87%	4.75%	4.43%	6.29%	5.10%	4.44%	6.76%
N-acetylputrescine	4.89%	2.07%	2.60%	3.65%	3.37%	1.68%	3.77%
N-acetylserotonin	9.15%	4.16%	3.53%	6.73%	6.30%	2.87%	6.92%
Nad	4.06%	10.76%	9.89%	8.64%	8.67%	0.81%	8.63%
Nicotinamide	3.17%	2.07%	1.89%	2.68%	2.43%	1.35%	2.78%
Phosphocholine	2.12%	2.30%	1.70%	2.01%	2.06%	0.52%	1.99%
Pipecolate	1.99%	2.17%	1.57%	1.88%	1.93%	0.50%	1.86%
Putrescine	3.46%	4.44%	3.52%	4.00%	3.84%	1.33%	4.07%
Pyridoxal	2.62%	4.66%	2.89%	3.41%	3.51%	1.00%	3.36%
Pyridoxine	2.18%	4.11%	3.14%	3.21%	3.25%	0.65%	3.18%
Pyruvate	8.56%	14.50%	1.70%	10.39%	9.84%	4.03%	10.63%
(5'-adenosyl)-methionine	17.07%	12.83%	12.75%	14.02%	14.27%	3.27%	13.90%
Sn-glycero-phosphocholine	5.73%	3.65%	2.98%	4.32%	4.26%	0.93%	4.36%
Spermidine	15.27%	6.73%	13.14%	13.03%	12.49%	4.51%	13.28%
Succinate	1.40%	2.45%	2.45%	2.35%	2.17%	1.10%	2.43%
Taurine	3.75%	4.50%	3.23%	3.79%	3.86%	0.89%	3.75%
Thiamine	5.10%	1.97%	1.00%	3.79%	3.28%	2.28%	4.00%
Tryptophan	2.18%	3.57%	2.70%	2.93%	2.89%	0.59%	2.95%
Uracil	5.29%	1.75%	5.10%	5.67%	4.38%	4.35%	6.17%
Urate	3.81%	3.21%	2.95%	3.35%	3.34%	0.35%	3.36%
Uree	4.58%	4.26%	4.43%	4.90%	4.42%	2.53%	5.10%
Uridine	2.51%	1.93%	2.03%	2.30%	2.18%	0.88%	2.35%
Uridine-monophosphate	13.64%	18.22%	21.25%	17.82%	17.74%	1.99%	17.85%
Xanthine	1.81%	2.14%	1.40%	1.77%	1.81%	0.48%	1.74%

**Internal Standards**		Within-day CV (%)			Between-day CV (%)	Within−day precision CV (%)	Between−day precision CV (%)	Intermediate precision CV (%)	**Recovery**
		**Day 01**	**Day 02**	**Day 03**					
17-Hydroxyprogesterone-d8	Start	0.96%	5.54%	8.67%	6.57%	5.78%	3.87%	6.96%	90.38%
(2,2,4,6,6,21,21,21-d8)	End	6.07%	4.03%	3.71%	4.44%	4.70%	0.57%	4.74%	
DL-Alanine-15N	Start	4.39%	4.42%	4.82%	8.36%	4.57%	5.00%	6.77%	92.43%
	End	8.30%	4.17%	4.54%	5.61%	5.67%	10.94%	12.32%	
L-Thyroxine-13C6	Start	2.66%	4.40%	7.10%	4.90%	3.25%	7.37%	8.05%	96.25%
	End	7.35%	6.80%	2.35%	5.58%	3.20%	14.29%	14.64%	
Pyruvic Acid 1-13C	Start	0.96%	5.54%	8.67%	6.57%	9.25%	11.77%	14.97%	47.33%
	End	6.07%	4.03%	3.71%	4.44%	4.81%	4.13%	6.34%	
Succinic Acid-2,2,3,3-d4	Start	4.39%	4.42%	4.82%	8.36%	2.56%	3.36%	4.22%	97.36%
	End	8.30%	4.17%	4.54%	5.61%	2.51%	3.30%	4.15%	

Supplementary Table S2: Example of metabolites validated and quantified from positive and negative ionisation modes during the validation process. The same validation procedure on 3 days published previously[1] has been used. The method was validated in terms of selectivity, repeatability, linearity, instrumental precision for all the metabolites detected and in term of extraction recovery for internal standards. Metabolites identification was possible using the MSMLS™ molecules library (IROA Technologies, Bolton, MA, U.S.A.) for mass spectrometry metabolomics. Identification criteria were as follow: an accurate m/z measurement (under 5 ppm), a perfect isotopic pattern, a RT drift lower than 5 s and/or 2 identical fragments (level of metabolite identifications: 1, Identified compounds)[2]. In other cases, molecules were named by their chemical formula and RT (level of metabolite identifications: 2, Putatively annotated compounds)[2]. Here, only the 76 molecules correctly identified at level 1[2] of the total 116 were summarised.

References

1. Kouassi Nzoughet, J. *et al.* A Nontargeted UHPLC-HRMS Metabolomics Pipeline for Metabolite Identification: Application to Cardiac Remote Ischemic Preconditioning. *Anal. Chem.* **89,** 2138–2146 (2017).
2. Sumner, L. W. *et al.* Proposed minimum reporting standards for chemical analysis: Chemical Analysis Working Group (CAWG) Metabolomics Standards Initiative (MSI). *Metabolomics* **3,** 211–221 (2007).
